# Scholarship Policies of International Students in Chinese Universities: A Brand Perception Perspective

**DOI:** 10.3389/fpsyg.2022.869171

**Published:** 2022-04-01

**Authors:** Nuo Wang

**Affiliations:** Financial Department, Changshu Institute of Technology, Changshu, China

**Keywords:** scholarship policies, international students, Chinese universities, higher education, brand perception

## Abstract

China has carried out a series of higher education reforms in the past decades. One of the most important parts of the reforms is the internationalization progress of Chinese universities. Despite being a developing country, China offers globally competitive scholarships to international students. However, surprisingly, little research has touched on how international students view China’s high scholarship policies, leaving an important and intriguing question underexplored. Therefore, this paper attempts to fill the literature gap by investigating international students’ brand perceptions of Chinese universities that provide high scholarships. Moreover, we reveal the process of their judgments through identifying the mediating role of perceived quality. A set of two experimental studies provide convergent support for these propositions. Theoretically, the findings of this paper contribute to the literature on higher education reforms, scholarship policies, and branding. In terms of practice, the research results offer implications to policy makers, education professionals, and students.

## Introduction

With the rapid development of its economy, massive reforms in higher education have taken place in China in recent decades, which have led to very significant and positive outcomes, largely enhancing the country’s higher education levels in many aspects. For instance, the proportion of education funds in GDP has stayed above 4% from 2012 to 2020. The total investment in education is 5.3 trillion yuan, of which 1.4 trillion have been invested in higher education, second only to compulsory education in 2020 (Ministry of Education of the People’s Republic of China, 2021b). By 2020, China’s higher education system had made significant advancements. The gross enrollment rate of higher education reached 54.4%, with the total number of students 41.83 million. To contrast, these numbers were 0.26% and 11.70 million in 1949, which clearly indicates China’s successful experience of building the world’s largest higher education system (Ministry of Education of the People’s Republic of China, 2019a, 2021a). In addition, Chinese universities have ranked increasingly higher in global universityb rankings in recent years. According to the latest 2022 Quacquarelli Symonds (QS) rankings, Tsinghua University, one of the best universities in China, ranked 17th in the world, is surpassing many well-known universities, including Cornell University, Columbia University, and Princeton University. The impressive performance of Tsinghua University is not a unique case. Indeed, six universities in mainland China scored in the top 100 in the world, whereas only two Chinese mainland universities were among the top 100 in 2010 (Quacquarelli Symonds, 2021).

In the process of higher education reform, one of the most important elements is the internationalization efforts of Chinese universities. To attract more international students to study in China, high scholarship policies were enacted. According to the Department of International Cooperation in the Ministry of Education, since 2001, the Chinese government has provided at least 11 scholarship programs for international students and scholars in China, including both full and partial scholarships (Ministry of Education of the People’s Republic of China, 2001). For example, one of the most well-known initiatives is the Chinese Government Scholarship Program, which has consisted of nine levels of scholarship ranging from 59,200 to 99,800 yuan per year since 2015 (Ministry of Education of the People’s Republic of China, 2015). As a reference, China’s GDP *per capita* was less than 47,000 yuan in 2015 (Central People’s Government, 2016). The Chinese Government Scholarship Program sponsored more than 420,000 international students to study in Chinese universities from 2003 to 2018 (Ministry of Education of the People’s Republic of China, 2019b). However, scholarships provided by the central government are only part of the scholarship system in China. The provinces and universities have also established scholarship programs for international students. Taken together, China has built a dynamic and fully integrated scholarship system, which is highly competitive worldwide.

However, although China’s high scholarship policies have had a huge impact, there has been sparse literature examining how international students cognitively view these policies, such as their brand perceptions, thus leaving an important research gap. Therefore, the present research investigates how China’s high scholarship offerings in higher education impact international students’ brand perceptions. We thus contribute to the literature in at least three ways. First, the extant literature mainly investigates the influence of a specific scholarship policy, while our findings are more general and not specific to a certain type of scholarship. Second, the previous research mainly focuses on the objective influences of the scholarship policies (e.g., economic returns and academic performance). In contrast, the present research sheds light on international students’ subjective perceptions of China’s high scholarship policies in higher education reform. Third, by investigating the brand of higher education reform and the internationalization of Chinese universities, this research seeks to extend the scope of brand research, contributing to the burgeoning stream of research on macro brands.

## Theoretical Framework

### The Role of Scholarship in Higher Education

Scholarship is one of the most important motivational stimuli in education. In an earlier paper by Brown et al. (1954), the researchers conducted a series of three studies on the motivational differences between high- and low-scholarship students, finding that students with low scholarship are characterized by activity delay, which manifests as a lack of decisiveness in taking action or a tendency to procrastinate. Finding of Ryan (1977) is different, however, in showing that scholarship athletes tend to have lower degrees of intrinsic motivation compared with non-scholarship athletes, which is consistent with famous notion of Deci (1971): the erosion function of external motivation (e.g., money) on internal motivation (e.g., interests). However, Amorose and Horn (2001) provided opposing evidence revealing that neither scholarship status nor time affects athletes’ levels of intrinsic motivation. Harkreader et al. (2008) found that Florida’s Bright Futures Program has significantly improved education quality by encouraging more high school students to take challenging courses, especially among low-income and minority students.

Much research focuses on the important role scholarship plays in higher education, in both the developed and developing countries. For example, through a test in an East Coast college in the United States, Van der Klaauw (2002) affirmed the importance of financial aid to attracting students, which is an effective instrument when competing with other colleges. According to Dynarski (2002), scholarship is an important type of financial aid that has a substantial impact on the college decisions of American high school graduates. Dynarski (2003) further found that the Social Security Student Benefit Program induced some students into college with a relatively low payoff before schooling but a relatively high payoff after schooling. Moreover, Chapman and Ryan (2005) examined the effects of the Higher Education Contribution Scheme (HECS) of Australia, which serves as the national income-contingent charge mechanism, finding that HECS results in strong growth in college participation, especially among students from middle-class families. Cornwell et al. (2005) investigated the Helping Outstanding Pupils Educationally (HOPE) Scholarship at Georgia State University. They showed that HOPE increases course withdrawals among freshmen, especially those who are on or below the scholarship-retention margin. Meanwhile, HOPE also significantly increases college students’ summer school credits. Goodman (2008) investigated the influence of the Adams Scholarship launched by the state of Massachusetts, showing that despite the relatively small monetary value, the scholarship leads to approximately 6% of its recipients choosing 4-year public colleges instead of private colleges. Using data on the 2001 cohort of the Gates Millennium Scholarship (GMS), Hu (2010) examined the relationship between scholarship and student engagement in college activities. The results from a survey indicated that receiving GMS awards increases the probabilities of attending 4-year colleges, while decreasing the probabilities of attending 2-year colleges. In addition, the GMS motivates college students to engage in more academic and social activities. Waddington and Berends (2018) analyzed the impact of the Indiana Choice Scholarship Program. They found that although school vouchers were designed to make better education resources more available to students in Indiana, the use of vouchers by low-income students to enter private schools does not indicate better academic performance.

### Internationalization of Chinese Universities

With the rapid development in its economy, higher education reforms have taken place in China over the past several decades. Similar to the China’s reform and opening-up policy that aims to fit into the world economic system, universities in China also actively engage in the globalization process. In terms of internationalization, we can follow two streams of literature to briefly summarize the related policies and their impacts.

The first stream regards how Chinese universities fit into the global academic community. According to statistics, from 1949 to 2019, the China Scholarship Council (CSC) sponsored more than 3 million Chinese students and scholars to study abroad (Yangcheng Evening News, 2021). Shimmi (2014) reported that Chinese visiting scholars are the largest group in all three categories of J-1 exchange visitors to the United States, including professors, research scholars, and short-term scholars. This trend is highly likely to maintain in the future. By interviewing Chinese visiting scholars who spent six to 12 months in Canadian universities, Qin and Jiang (2015) showed that overseas experience fosters a number of positive outcomes for the visiting scholars. For example, they utilize their Canadian experience to enhance their teaching levels, initiate new research projects, and publish more academic papers. Xue et al. (2015) observed and interviewed 15 Chinese visiting scholars during their stay in the United States academic community, exploring their communication strategies in the process of academic socialization. The results indicate that United States hosting organizations do not provide enough support for the visitors because of the defects within the visiting programs.

The second area pertains to Chinese universities’ efforts to attract more international students. To date, more than 492,185 students from 196 countries have studied in mainland China, in over 1,000 higher education institutions (University World News, 2021). Dong and Chapman (2008) found that international students in China are generally satisfied with their experiences. Moreover, since the Chinese Government Scholarship Program offers the opportunity to international students to study free at Chinese colleges, international students are positive about the future impact of the program to help build friendships between China and their home countries. Wen et al. (2014) examined a sample of 1,674 international students in China, finding that students who have more frequent social interactions with residents in the host countries are likely to receive more social support and are better poised for sociocultural adaptation. Based on a questionnaire survey and in-depth interviews, Ding (2016) examined the reasons why more and more international students favor China. Given the unique features of the Chinese culture, the country’s rapid economic growth, and friendly scholarship policies, China has become an important market for international students. However, to maintain sustainable growth in the international student market, China needs to enact more globally attractive policies and further enhance international students’ satisfaction with their learning and living experiences. Jiani (2017) explored why a growing number of students choose China as their study abroad destination, showing that one of the central motivations is China’s excellent development potential of economy. Another interesting finding is that the proportion of descendants of overseas Chinese who apply for Chinese universities is growing. These individuals wish to restore their cultural identities by studying in China.

In sum, as the only developing country using major transformations in tertiary educational delivery as a development strategy, China is interested in confronting the challenge of its population size and seized this economic growth opportunity. The process of education transformation can have major impacts on China, as well as on the global economy and global educational structure (Li et al., 2011).

### Scholarship and Students’ Brand Perceptions

Branding serves as one of the central elements in many business activities, such as marketing (McDowell, 2006). A large body of literature has verified the important role of branding from various perspectives. From a strategic point of view, Wong and Merrilees (2007) argued that an international branding strategy can help enhance a firm’s brand and financial performance abroad. Furthermore, Brodie (2009) examined the importance of branding in the context of service, emphasized the necessity of understanding the nature of value propositions, and developed a new theoretical framework illustrating how a brand can function both as an entity and a process. Based on the Strategic Management of Service Brand Relationships model, Tsai (2011) documented that service brand commitment and service brand love serve as the major antecedents of service brand loyalty.

It is worth noting that an emerging stream of literature on branding has tended to view brands more broadly. For example, considering the anthropomorphic property of brands (Aggarwal and McGill, 2012), personal brands have received increasing research attention. Building on the theory of social identity and brand personality, Carlson and Donavan (2013) developed and tested a model in which perceptions of human brands affect consumers’ cognitive identifications. For example, empirical analyses suggest that consumers view athletes as human brands with human personalities. Huang et al. (2015) tested the factors that might affect the loyalty of idol brands. Different from previous research, the researchers suggested that the association between the physical appearance of vanity and the attachment to idol brands is insignificant. The importance of personal brands is not limited to celebrities, however. Luca et al. (2015) defined a personal brand as the goal toward making one’s expertise or uniqueness known in a certain social circle or for a particular cause. Similar to a successful commercial brand, an impactful personal brand needs a beautiful cover, as well as a solid content. The notion of brand can even reach the national level. Rojas-Méndez (2013) proposed the concept of the nation brand molecule (NBM), which consists of seven dimensions, including economy, tourism, geography and nature, culture and heritage, society, science and technology, and government.

Because of processing complexity and information asymmetry, people tend to rely on mental shortcuts to form their impressions about brand images (Manes and Tchetchik, 2018). As a type of psychological rating, cognitive bias is a common place in brand evaluations. Thorndike (1920) defined the halo effect as the influence of individual attributes evaluations on the global evaluation of a given object, especially an unfamiliar one. Influenced by the halo effect, people often judge a person or a product overall as positive if they observe it has one or two obvious advantages, which also occurs frequently when evaluating brands (Leuthesser et al., 1995), even though they may not be consciously aware of this perception (Nisbett and Wilson, 1977).

On the basis of the above theoretical foundations, we propose that Chinese universities’ high scholarship policies enhance brand perceptions among international students. This is because high scholarship functions as a strong and highly visible cue to international students; in other words, under the influence of the halo effect, they form good impressions toward higher education in China, which leads to positive brand perceptions. This mental process is in line with the country-of-origin theory, which describes a cognitive cue based on which consumers generate beliefs regarding product attributes, such as, quality (Bilkey and Nes, 1982). In a meta-analysis paper by Verlegh and Steenkamp (1999), the effect of country-of-origin on the perceived quality of products is very strong. Moreover, the underlying reason for the country-of-origin effect is economic development, such that consumers generally believe that products made in wealthier countries have better performance in related dimensions (e.g., quality and taste) as well. In the case of scholarship policies, high scholarship is a clear signal of economic strength, which, in turn, indicates competitive educational levels (Jorgenson and Fraumeni, 1992), resembling a country-of-origin effect in brand perceptions. For instance, consumers are usually willing to pay more for a made-in-Italy luxury product than for another one with similar attributes in all other dimensions but made in a developing country (Cheah et al., 2016). In a similar vein, thanks to its high scholarship policies, study in China is viewed as a well-established brand in the overseas study market.

Ownership plays an important role in the psychological information process is worth noting. The endowment effect, a classic theory in behavioral economics, proposes that people demand much more to give up an object compared with the price they are willing to pay to acquire it (Thaler, 1980). Similarly, Beggan (1992) found that mere ownership of an object can create a firm psychological association between the owner and the object, which, in turn, leads the owner to regard the object as a social entity; this is termed the “mere ownership effect.” In an interesting paper by Dai and Hsee (2013), they revealed how ownership profoundly affects people’s judgments and decision making. For example, ownership alters the relationship between a person’s fullness and the perceived size of a cake. Specifically, in ownership, one perceives the cake to be smaller when hungry than when satiated. On the contrary, without ownership, the person perceives the cake to be larger if hungry than if satiated.

However, we believe that ownership hardly influences the impact of scholarship on brand perceptions. This is because brand perceptions are much more ambiguous than food judgments. As the General Evaluability Theory states, knowledge is one of the key determinants in the evaluation process. The more knowledge one possesses toward an object, the more precise the evaluating results will be (Hsee and Zhang, 2010). Inferring the size of a cake is simple, as most people have a clear understanding of food. In contrast, non-educational professionals can hardly accumulate much knowledge of universities in an emerging country (e.g., China).

*Hypothesis 1: China’s high scholarship policies enhance its brand perceptions among international students, regardless of ownership conditions.*

### The Mediating Role of Perceived Quality

We propose that perceived quality mediates the relationship between scholarship policies and brand perceptions. Following the logic of the causal chain, the evidence is 2-fold. First, high scholarship promotes quality perceptions. In line with the halo effect and the country-of-origin theory, people believe that universities providing high scholarship are more competent (e.g., higher academic impacts, better social reputations, etc.) and thus more able to offer higher educational quality. Second, quality perceptions positively contribute to brand images. Previous research has verified that quality is one of the most proximal predictors of brand perceptions and loyalties (Chi et al., 2009; Shanahan et al., 2019). Thus, when people consider a study destination to be of high quality, positive brand perceptions are generated.

*Hypothesis 2: The effect of scholarship policies on brand perceptions is mediated by perceived quality.*

## Studies Overview

We designed and conducted a set of two studies to provide evidence for the hypotheses. Considering the advantage of behavioral experiments in clarifying causal relationships, the studies adopt an experimental approach. In Study 1, we tested the effect of China’s high scholarship on American subjects’ brand perceptions (H1), as well as the mediating role of perceived quality (H2). To enhance the robustness of the findings, Study 2 made three significant changes while examining the two hypotheses, including recruiting British subjects, adopting another manipulation method, and using a new and more objective measure of brand perceptions.

## Study 1

### Method

#### Participants and Design

One hundred and nine participants (66 males, one participant did not provide gender information; *M_age_* = 39.46 years, *SD* = 11.72) recruited from Mturk (DellaVigna and Pope, 2018; Wang et al., 2020) took part in our study in exchange for monetary payment. They were randomly assigned to one of the four conditions in a 2 (scholarship: high vs. low) × 2 (ownership: yes vs. no) between-subjects design.

#### Procedure

All participants were asked to engage in an imaginary task. To manipulate ownership states, we asked the participants in the with (without) ownership condition to imagine that a university in China gave them an offer (they had a study-abroad plan and noticed a university in China). To manipulate the scholarship levels, participants in the high (low) scholarship condition were told that the university could provide them a very high (low) scholarship, among the top (bottom) 10% globally. Subsequently, as the manipulation check of scholarship levels, all participants indicated the extent to which they agreed with the following statement: “The University mentioned offers high scholarship.” Their responses were provided on a five-point scale (1 = *strongly disagree*; 5 = *strongly agree*).

The participants then proceeded to the measurements of the mediator and dependent variable. Specifically, they responded to a two-item scale (*α* = 0.92) measuring perceived quality adapted from Boris et al. (2004) on a five-point Likert scale (1 = *strongly disagree*, 5 = *strongly agree*). The items are: “I think this is a pretty good university.” and “This is a competent university.” Afterward, the participants indicated their brand evaluations for the university in the scenario on three five-point items (*α* = 0.94). The scale is adapted from Mitchell and Olson (1981) and composed of three semantic differential items (i.e., positive–negative, good-bad, and likable-dislikeable), which were averaged to create an index of brand perceptions. Finally, after reporting demographic information, the participants were thanked and debriefed.

### Results

#### Manipulation Check

As expected, a two-way ANOVA on perceived scholarship only yielded a main effect of scholarship, such that participants in the high scholarship condition reported higher perceptions of scholarship compared with those in the low scholarship condition [*M_high scholarship_* = 4.69, *SD* = 0.11; *M_low scholarship_* = 1.26, *SD* = 0.13; *F*(1, 105) = 415.19, *p* < 0.001]; neither the main effect of ownership nor the interaction effect emerged (*F*_s_ < 3.00, *p*_s_ > 0.09). These results indicate that our manipulation of scholarship levels was successful.

#### Brand Perceptions

**Figure 1:**
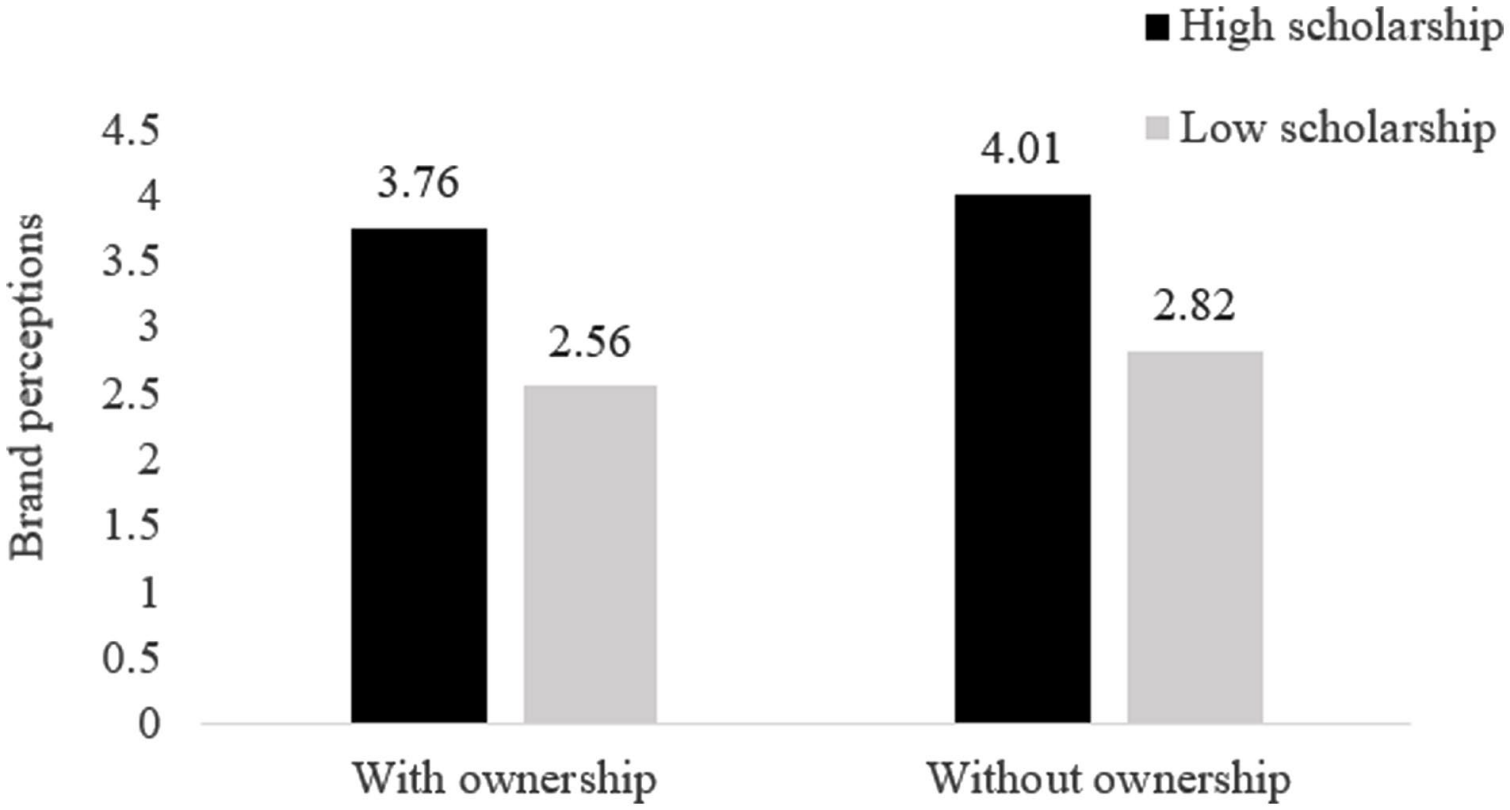
The effect of scholarship policies and ownership levels on brand perceptions (Study 1).

We conducted another two-way ANOVA with brand perceptions as the dependent variable and scholarship and ownership manipulation as the independent variables (see Figure 1). In line with our expectation, only a significant main effect of scholarship emerged [*F*(1, 105) = 51.94, *p* < 0.001]; the participants in the high scholarship condition made higher brand evaluations for the university in the scenario than those in the low scholarship condition (*M_high scholarship_* = 3.89, *SD* = 0.11; *M_low scholarship_* = 2.69, *SD* = 0.13). The results showed no significant effect of either ownership or the interaction term (*F_s_* < 2.33, *p_s_* > 0.13).

#### Perceived Quality

A two-way ANOVA on perceived quality showed that the participants in the high scholarship condition also expressed better quality inferences for the school (*M* = 3.83, *SD* = 0.11) compared with those in the low scholarship condition [*M* = 2.57, *SD* = 0.14, *F*(1, 105) = 50.03, *p* < 0.001]. No other effects were significant (*F_s_* < 1.71, *p_s_* > 0.19).

#### Mediation Analyses

A moderated mediation analysis (PROCESS Model 7; Hayes, 2013) using 1,000 samples supported the indirect effect of scholarship policies on brand perceptions through perceived quality. Specifically, the results revealed that the mediation of perceived quality remained significant regardless of the levels of ownership (without ownership: *b* = 1.15, *SE* = 0.21, and 95% CI = 0.75–1.55; with ownership: *b* = 0.79, *SE* = 0.21, and 95% CI = 0.39–1.22). In other words, the effect of scholarship policies on brand perceptions was not affected by ownership levels (*b* = −0.36, *SE* = 0.27; and 95% CI = −0.94–0.16).

### Discussion

The results of this study provide evidence that China’s high scholarship policies enhance brand perceptions among international students, regardless of ownership states (H1). In addition, this effect is shown to be mediated by perceived quality (H2). However, the samples were only recruited from one country in Study 1, and the manipulation methods and measurement of the dependent variables remained to be triangulated, which led us to design and conduct Study 2.

## Study 2

### Method

#### Participants and Design

One hundred and twenty-four participants (46 males, one participant did not provide gender information; *M_age_* = 33.58 years, *SD* = 11.56) recruited from Prolific (Palan and Schitter, 2018; Wang et al., 2022) took part in this study in exchange for monetary payment. They were randomly assigned to one of the four conditions in a 2 (scholarship: high vs. low) × 2 (ownership: yes vs. no) between-subjects design.

#### Procedure

All participants were asked to engage in an imaginary task. To manipulate ownership states, we followed Dai and Hsee (2013) and asked participants in the with (without) ownership condition to imagine they were studying abroad in China (one of their formal classmates was now studying abroad in China). We manipulated the scholarship policies following the same procedure we used in Study 1. Afterward, the participants completed the manipulation check questions regarding perceived scholarship using the same item as in Study 1.

The participants then proceeded to the measurements of the mediator and dependent variable. Specifically, perceived quality was measured using the same scale (*α* = 0.91) as in Study 1. Afterward, the participants were asked to rate their perceived ranks of the university, adapted from Geuna and Martin (2003), which includes five items (*α* = 0.94) of five dimensions (i.e., academic research, faculty resources, student excellence, alumni giving, and social reputation). Previous research suggests that ranking serves as a valid measurement of brand image perceptions (Driesener and Romaniuk, 2006). Again, they responded on a five-point scale (1 = *bottom globally*, 5 = *top globally*). Finally, after reporting demographic information, the participants were thanked and debriefed.

### Results

#### Manipulation Check

As expected, a two-way ANOVA on perceived scholarship only yielded a main effect of scholarship, such that participants in the high scholarship condition reported higher perceptions of scholarship compared with those in the low scholarship condition [*M_high scholarship_* = 4.18, *SD* = 0.15; *M_low scholarship_* = 1.39, *SD* = 0.13; *F*(1, 120) = 197.95, *p* < 0.001]. Neither the main effect of ownership nor the interaction effect emerged (*F*_s_ < 1.83, *p* > 0.18). These results indicate that our manipulation of scholarship levels were successful.

#### Brand Perceptions

**Figure 2:**
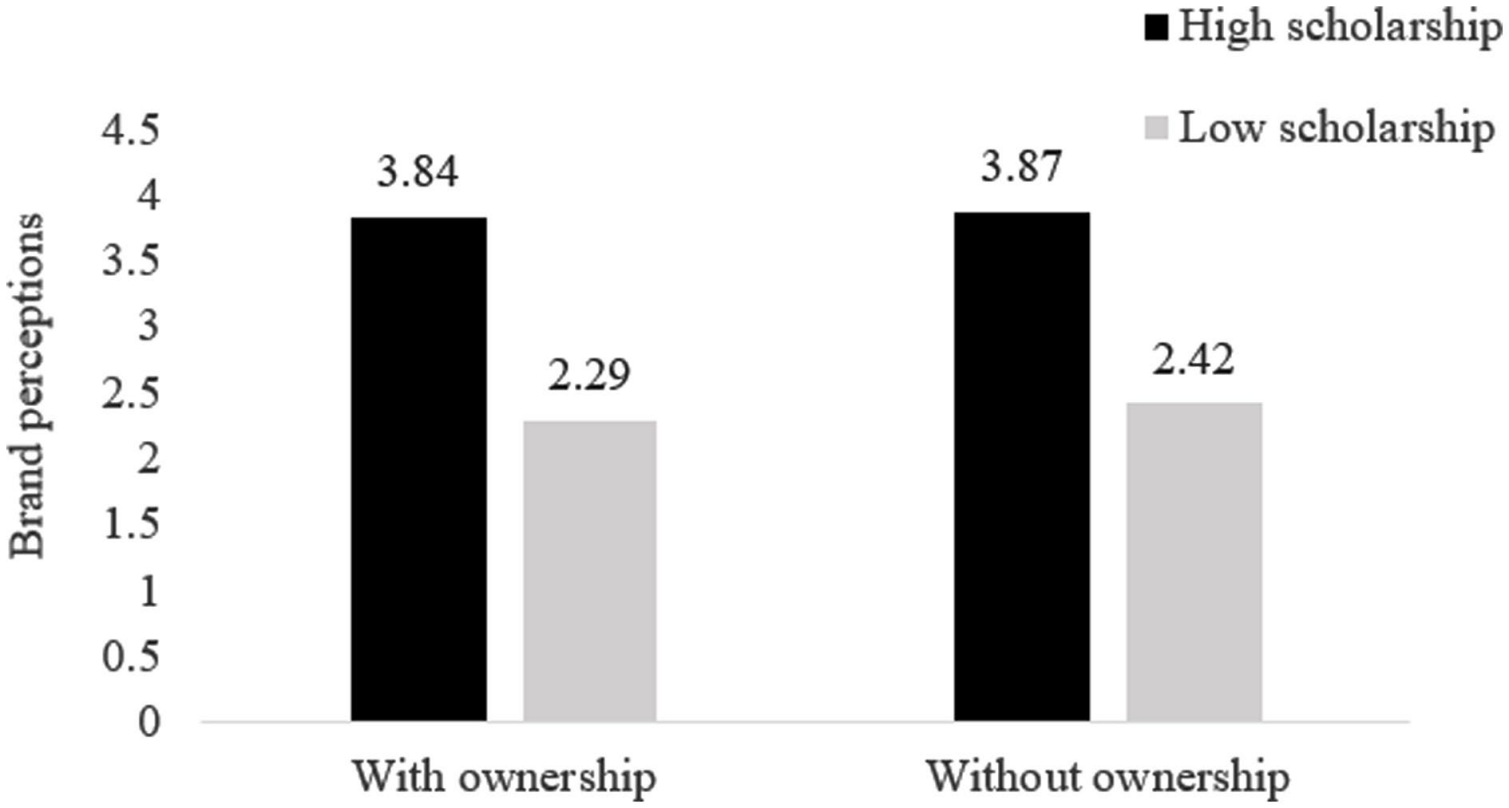
The effect of scholarship policies and ownership levels on brand perceptions (Study 2).

We have conducted another two-way ANOVA with brand perceptions as the dependent variable and scholarship and ownership manipulation as the independent variables (see Figure 2). In line with our expectation, only a significant main effect of scholarship emerged [*F*(1, 120) = 159.28, *p* < 0.001]; the participants in the high scholarship condition made higher brand evaluations for the university in the scenario than those in the low scholarship condition (*M_high scholarship_* = 3.86, *SD* = 0.08; *M_low scholarship_* = 2.36, *SD* = 0.08). The results showed no significant effect of either ownership or the interaction term (*F_s_* < 0.44, *p_s_* > 0.50).

#### Perceived Quality

A two-way ANOVA on perceived quality showed that participants in the high scholarship condition also expressed better quality inference for the school (*M* = 3.84, *SD* = 0.12) compared with those in the low scholarship condition [*M* = 2.40, *SD* = 0.10, *F*(1, 120) = 86.13, *p* < 0.001]. No other effects were significant (*F_s_* < 1.28, *p_s_* > 0.26).

#### Mediation Analyses

A moderated mediation analysis (PROCESS Model 7; Hayes, 2013) using 1,000 samples supported the indirect effect of scholarship policies on brand perceptions through perceived quality. Specifically, the results revealed that the mediation of perceived quality remained significant regardless of the levels of ownership (without ownership: *b* = 0.58, *SE* = 0.17, 95% CI = 0.26–0.91; with ownership: *b* = 0.74, *SE* = 0.17, 95% CI = 0.43–1.07). In other words, the effect of scholarship policies on brand perceptions were not affected by ownership levels (*b* = 0.16, *SE* = 0.14; 95% CI = −0.12–0.44).

### Discussion

Through recruiting a different sample and adopting a new manipulation method as well as dependent variable measurement, Study 2 replicated and thus verified the robustness of the findings in Study 1. Taken together, these two studies provide convergent and strong support for the research hypotheses.

## General Discussion

In this research, we proposed and found that China’s high scholarship policies enhance its brand perceptions among international students, regardless of ownership conditions. This effect occurs because high scholarship boosts quality inferences, which, in turn, promotes brand images. Two experimental studies provide convergent support for these notions. In Study 1, we demonstrated that China’s high scholarship can elevate American subjects’ brand perceptions of Chinese universities, and that the underlying mechanism of this effect is perceived quality. To enhance the robustness of the findings, Study 2 recruited British subjects, adopted another manipulation method, and used a new measure of brand perceptions. As the results replicated those in Study 1, the two studies constitute a strong empirical package that supports the hypotheses.

### Theoretical Contributions

The present paper contributes to the related literature in at least three ways. First, the extant literature mainly investigates the influence of a specific scholarship policy, for instance, the Social Security Student Benefit Program (Dynarski, 2003), the HECS of Australia (Chapman and Ryan, 2005), the HOPE Scholarship of Georgia (Cornwell et al., 2005), the Adams Scholarship of Massachusetts (Goodman, 2008), the Gates Millennium Scholarship (Hu, 2010), and the Indiana Choice Scholarship Program (Waddington and Berends, 2018). Our findings are more general, as we reveal that China’s high scholarship can boost international students’ brand perceptions, which is not specific to certain types of scholarship.

Second, previous research focuses on the objective influences of scholarship policies, such as college and course preferences (Dynarski, 2002; Cornwell et al., 2005; Harkreader et al., 2008), economic returns after education (Dynarski, 2003), enrollment rates (Chapman and Ryan, 2005), college activity engagements (Hu, 2010), and academic performance (Waddington and Berends, 2018), while the downstream consequences in terms of image perceptions regarding overseas study destinations have long been ignored (Lee, 2014). Consequently, the present research adds to the literature on international students’ psychological responses to China’s high scholarship policies in higher education reform (Dong and Chapman, 2008; Wen et al., 2014).

Third, by introducing the concept of brand to higher education reform and the internationalization of Chinese universities, this research has extended the scope of brand research to the education and even national level, thus enriching the burgeoning stream of research on broader brands, such as, personal brands (Carlson and Donavan, 2013; Huang et al., 2015; Luca et al., 2015) and nation brands (Rojas-Méndez, 2013).

### Practical Implications

In terms of practice, the findings in this research are instructive for government policy makers, education professionals, and students. First, for emerging countries that would like to build impactful education brands, monetary investment may still serve as one of the most effective ways. In the long run, the brand construction cost can be economically rewarded by the tuitions of international students and by the international students’ local consumption, which contributes to GDP. Second, education professionals can learn the criteria adopted by students in evaluating overseas study destinations, and based on the acquired knowledge, can design effective service strategies. Third, for students who plan to receive higher education abroad, our findings point out the necessity of considering study destinations more holistically. Halos (e.g., high scholarship) can be easy shortcuts to draw conclusions, but may also turn into traps in decision making.

### Limitations and Future Research

This research has several limitations that indicate potential directions for future research. First, China recently released a series of university scholarship policies mainly directed at developing countries, such as the Belt and Road Initiative Scholarship. Given that the present paper only collected data from developed countries, future research could recruit more samples from developing countries to examine the cross-cultural validity of the findings. Second, though we have identified perceived quality as the underlying factor, alternative explanations may still exist. For instance, drawing on the social reciprocity theory (Carpenter and Matthews, 2004), one can posit that the reason why Chinese universities’ high scholarship leads to positive brand evaluations is that people simply offer social reciprocity to universities that present them high incentives. Future research could adopt the Implicit Attitude Test (Rooth, 2010) to rule out this potential confounding. Third, the effect of scholarship policies on brand perceptions is shown to be robust regardless of ownership states, but we cannot eliminate the existent possibility of boundary conditions, under which the effect is mitigated or even reversed. In sum, it will be theoretically contributive to investigate different moderators in future research. Finally, though we used multisource samples in this research, the participants recruited from online panels may not be the group of students who have the most urgent needs to study abroad. Future research can use more appropriate samples for empirics to further enhance external validity.

## Data Availability Statement

The raw data supporting the conclusions of this article will be made available by the authors, without undue reservation.

## Ethics Statement

Ethical review and approval was not required for the study on human participants in accordance with the local legislation and institutional requirements. Written informed consent for participation was not required for this study in accordance with the national legislation and the institutional requirements.

## Author Contributions

The author confirms being the sole contributor of this work and has approved it for publication.

## Conflict of Interest

The author declares that the research was conducted in the absence of any commercial or financial relationships that could be construed as a potential conflict of interest.

## Publisher’s Note

All claims expressed in this article are solely those of the authors and do not necessarily represent those of their affiliated organizations, or those of the publisher, the editors and the reviewers. Any product that may be evaluated in this article, or claim that may be made by its manufacturer, is not guaranteed or endorsed by the publisher.
